# Iatrogenic Hypoxemia and Atrial Septal Defect Due to Electrical Storm Ablation After Left Ventricular Assist Device: A Case Report

**DOI:** 10.7759/cureus.39418

**Published:** 2023-05-23

**Authors:** Ryan Wang, Darcy J Mainville, Alexandra Vacaru, Ioana Pasca

**Affiliations:** 1 Anesthesiology, Loma Linda University Health, Loma Linda, USA; 2 Critical Care Medicine, Loma Linda University Health, Loma Linda, USA; 3 Anesthesiology, Riverside University Health System, Moreno Valley, USA

**Keywords:** atrial septal defect, implantable cardiac defibrillator, left ventricular assist device, transesophageal echocardiogram, ventricular tachycardia, arterial blood gas, hypoxemia, catheter ablation

## Abstract

A 59-year-old male with an implantable cardiac defibrillator, left ventricular assist device, and refractory ventricular tachycardia presented with hypoxemia due to a post-ablation iatrogenic atrial septal defect. Left ventricular assist devices generate pressure gradients that may exacerbate intracardiac shunts and can precipitate significant hypoxemia.

## Introduction

Electrical storm is hallmarked by recurrent episodes of ventricular tachycardia (VT), ventricular fibrillation, or multiple appropriate shocks from an implantable cardiac defibrillator (ICD) over a short period, typically 24 hours. Management of electrical storm is challenging, and primary intervention involves the identification and correction of precipitating factors, such as myocardial ischemia as well as arrhythmia suppression with antiarrhythmics and beta-blockers. Refractory arrhythmias may warrant catheter ablation [1,2]. Although mechanical circulatory support may be used in the management of electrical storm, ventricular arrhythmias may occur after left ventricular assist device (LVAD) implantation. One study showed that 23% of patients developed at least one ventricular arrhythmia after LVAD placement. Approximately 4% of LVAD patients experience five or more episodes of arrhythmia events in the immediate post-operative period [3]. Catheter ablation is not only feasible with LVAD but is safe and effective. Even in patients with incessant VT, catheter ablation reduces VT recurrence [3,4]. We present the management of a patient with LVAD and electrical storm and describe the development of refractory hypoxemia after catheter ablation. Health Insurance Portability and Accountability consent was obtained from the patient for publication of this study.

## Case presentation

A 59-year-old male with acute left anterior descending ST-elevation myocardial infarction, failed percutaneous coronary intervention, left ventricular ejection fraction of 20%, and recurrent VT was transferred to our institution for a higher level of care. After a failed percutaneous coronary intervention, he developed persistent monomorphic VT and recurrent ICD shocks refractory to lidocaine and amiodarone.

He was evaluated for LVAD implantation for ischemic cardiomyopathy and refractory VT. Concurrent epicardial cryoablation was planned to address recurrent ventricular arrhythmias. The HeartMate 3 LVAD (Chicago, IL: Abbott) was implanted, and cryoablation was performed adjacent to the LVAD core. Post-operatively, he was started on procainamide up to a maximum of 4 mg/min, but he continued to experience recurrent episodes of VT. Electrophysiology recommended endocardial catheter ablation.

Procedure

Left ventricular access was obtained via a standard transseptal approach. After initial transseptal access was obtained, extensive ablation of the left and right ventricular aspects of the interventricular septum was performed. After ablation, the clinical VT was no longer inducible.

**Figure 1:**
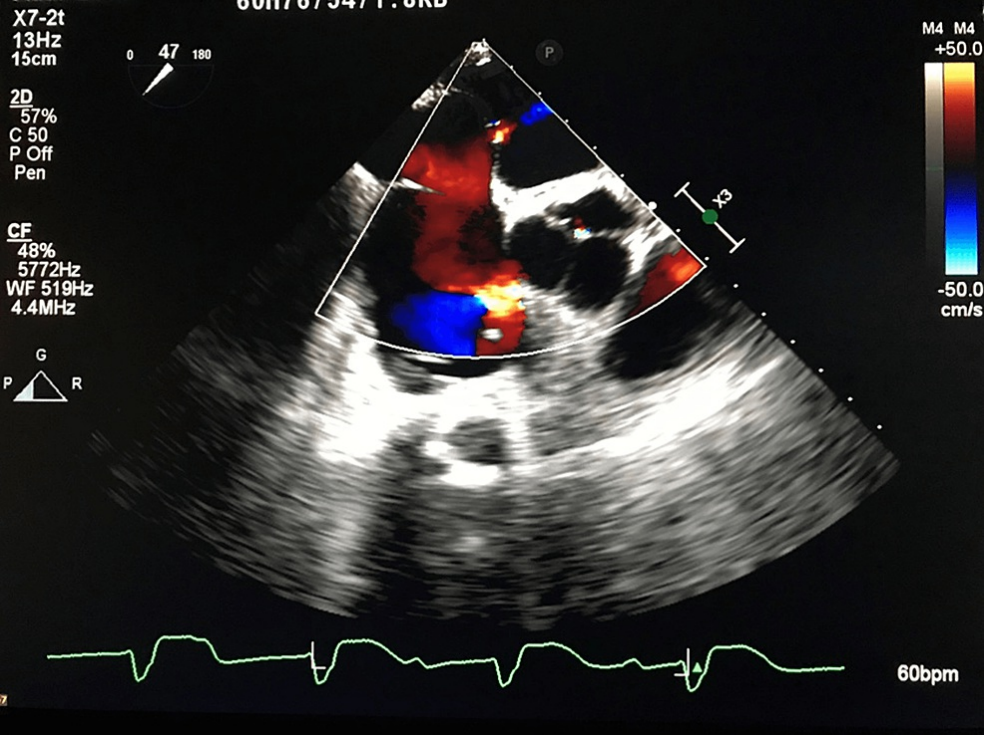
TEE visualization of ASD. TEE: transesophageal echocardiogram; ASD: atrial septal defect

Post-procedure, he remained intubated on mechanical ventilation with a large alveolar-arterial gradient and a P/F ratio of 93 on 100% FiO_2_. His arterial blood gas (ABG) on 100% FiO_2_ was pH 7.40, PaCO_2_ 33 mmHg, PaO_2_ 94 mmHg, and HCO_3_ 19.9 mEq (Table 1). A bedside transesophageal echocardiogram (TEE) demonstrated a 3 mm transseptal puncture atrial septal defect (ASD) with moderate right to left shunt across the defect (Figure 1). His hypoxemia was attributed to the right-left shunt and closure of the defect was advised.

**Table 1:** Arterial blood gas pre- and post-ASD closure. ASD: atrial septal defect; BE: base excess

Variables	pH	PCO_2_ (mmHg)	PO_2_ (mmHg)	HCO_3_ (mEq)	BE (mmol/L)	Lactate (mmol/L)	FiO_2_
Pre-ASD closure	7.40	33	94	19.9	3.7	1.0	100%
Post-ASD closure	7.34	39	198	20.6	4.1	1.0	100%

He returned to the cardiac catheterization laboratory for ASD closure 48 hours post-ablation. A 30-mm Cardioform device (Newark, DE: W.L. Gore and Associates) was deployed under fluoroscopy and TEE guidance, and the ASD was closed. Prior to device deployment, he was on 100% FiO_2_ with a PaO_2_ of 100 mmHg. After ASD closure, his PaO_2_ increased to 200 mmHg on 100% FiO_2_. His ABG with the same ventilator settings on 100% FiO_2_ after device deployment and closure of his ASD was pH 7.34, PaCO_2_ 39 mmHg, PaO_2_ 198 mmHg, and HCO_3_ 20.6 mEq (Table 1). His right atrial pressure was 13 mmHg pre-device placement and 14 mmHg post-device placement. Left atrial pressure was 8 mmHg. His ICD generator was also changed as the battery was depleted from delivery of recurrent shocks.

After ASD closure, hypoxemia resolved and arterial saturation improved. He was extubated to a high-flow nasal cannula 48 hours later. His hemodynamic status continued to improve, and he was discharged to acute rehabilitation.

## Discussion

Predominant strategies for the management of electrical storm include antiarrhythmic medical therapy, beta-blockers, and catheter ablation [1,5]. Some studies have demonstrated refractory electrical storm managed with mechanical circulatory support, but VT may also be exacerbated by LVAD placement. LVAD implantation may be associated with electrolyte changes, repolarization abnormalities, myocardial scar, and ventricular chamber collapse, which may precipitate arrhythmia [6].

When electrical storm is refractory to medical therapy, endocardial ablation may be indicated. Although catheter ablation has been described in patients with LVAD, the complications uniquely associated with catheter ablation in the LVAD population have not [3,4]. In a limited series, Sacher et al. described the outcomes of catheter ablation in 34 patients with LVAD. Among these, one patient developed cardiogenic shock with acidosis, one experienced a transient ischemic attack, one experienced a stroke eight days after ablation, and one required a blood transfusion with two units of packed red blood cells for a groin hematoma [7].

The patient presented suffered from refractory electrical storm after LVAD implantation and epicardial ablation. An endocardial approach was required to address the residual arrhythmogenic foci. Transseptal puncture is routinely performed for left ventricular access, and the small residual ASD usually self-seals. Persistent iatrogenic ASD has been described after pulmonary vein isolation for atrial fibrillation [8,9]. However, the majority of iatrogenic ASDs are associated with inconsequential left-to-right shunt and usually close within 12 months [10]. A few studies have described the closure of ASD after LVAD [11,12]. However, our case is one of the first to describe refractory hypoxemia from an iatrogenic ASD in a patient with LVAD after catheter ablation. 

Persistence of a right-to-left shunt requires a significant pressure gradient between the right and left atrium. The LVAD offloads the left ventricle and decreases left atrial pressure. When unloading of the left side of the heart is coupled with elevated right atrial pressures, the pressure gradient may be sufficient to support a persistent right-to-left shunt. Our patient likely had elevated right atrial pressure from moderate tricuspid regurgitation. Furthermore, implantation of an LVAD significantly increases the workload of the right ventricle. In patients with cardiomyopathy and reduced cardiac output, the right ventricle has acclimated to a lower cardiac output state. When an LVAD is implanted, the right ventricle is forced to adapt to much higher cardiac output and may precipitate right ventricular dysfunction and additional increases in right atrial pressure [13]. Transseptal puncture requires careful consideration in the LVAD population, persistent hypoxemia should raise concern for the presence of a right-to-left shunt [14]. We advocate for continuous oximetry and careful attention to oxygen requirements post-procedure. Consider TEE evaluation in patients with unexplained hypoxemia.

## Conclusions

Endocardial catheter ablation is a safe and effective treatment for electrical storm. Although mechanical circulatory support has been used to reduce the burden of arrhythmia, LVAD may also be associated with persistent ventricular arrhythmias. When a transseptal puncture is performed in a patient with an LVAD, conditions are optimal to support persistence of a right-to-left shunt, and we recommend post-ablation TEE to evaluate for intracardiac shunts. Right-to-left shunts may cause significant hypoxemia and warrant emergent closure. LVAD implantation dramatically alters intracardiac pressures and can exacerbate or even reverse intracardiac shunts.
